# Co-existence of Sarcina Organisms and Helicobacter pylori Gastritis/Duodenitis in Pediatric Siblings

**Published:** 2013-09-05

**Authors:** Jennifer L. Sauter, Suresh K. Nayar, Paige D. Anders, Michael D’Amico, Kelly J. Butnor, Rebecca L. Wilcox

**Affiliations:** 1 Department of Pathology and Laboratory Medicine, The University of Vermont/Fletcher Allen Health Care, Burlington, VT, USA; 2 Department of Pathology, Johns Hopkins School of Medicine, Baltimore, MD, USA; 3 Department of Pediatric Gastroenterology, The University of Vermont/Fletcher Allen Heath Care, Burlington, VT, USA

**Keywords:** Sarcina, Helicobacter pylori, Gastric outlet obstruction, Duodenitis, Gastritis

## Abstract

*Sarcina* are gram-positive anaerobic bacteria found to be associated with delayed gastric emptying and gastric outlet obstruction. We describe two cases of *Sarcina* co-existing with *Helicobacter pylori* organisms in pediatric siblings presenting within four months of each other with pyloric obstruction secondary to severe gastritis/duodenitis. The co-existence of Sarcina and *Helicobacter pylori* has not, to our knowledge, been previously reported. Its characteristic tetrad packeted morphology permits *Sarcina* to be readily identified on routine sections. Detection of these organisms in gastric biopsies should prompt consideration of gastric outlet obstruction and/or delayed gastric emptying as a possible etiologic factor.

## Introduction

*Sarcinaventriculi (S. ventriculi)* are gram-positive, obligate anaerobic bacteria first documented in the human gastrointestinal tract in 1842 [1–4]. Latin for “package” or “bundle,” Sarcinacocci undergo fission in three planes, perpendicular to one another, resulting in their characteristic packeted morphology, often in tetrads but occasionally in cubes of eight [3,5,6]. In addition, *S. ventriculi* has a thick (150 to 200 nm) fibrous layer on the outer surface of its cell wall composed mostly of cellulose [1,3]. Given these unique conjoining features—tetrad packets with a thick cellulose-dominant wall—it is not surprising that these organisms were initially thought to be vegetable matter [3].

The purpose of this article is to report an unusual occurrence of pediatric siblings with metachronous presentations of severe *Helicobacter pylori (H. pylori)* gastritis/duodenitis with coexisting Sarcina. Only rare pediatric cases of human disease associated with *Sarcina* organisms have been documented [7]. To our knowledge, these are the first cases of *Sarcina* organisms co-existing with *H. pylori.*

## Report of a Case

### Clinical history

**Figure 1:**
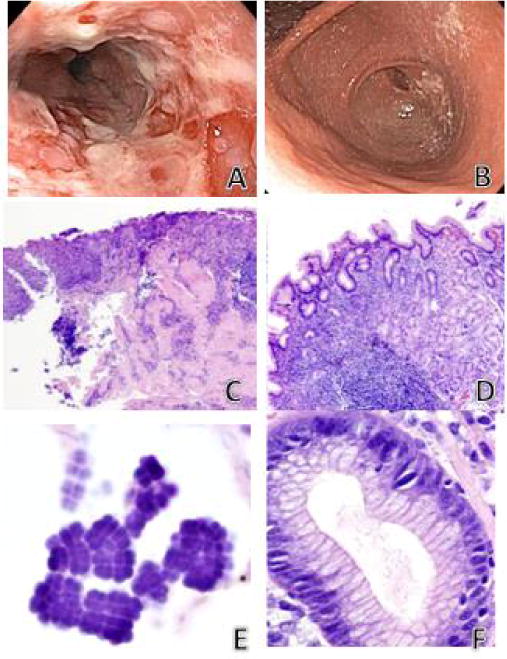
Brother’s endoscopic images. A) Severe erosive esophagitis in the distal third of esophagus. B) Marked narrowing (pinpoint lumen) of pylorus. Sister’s endoscopic images (four months later) were morphologically similar however there was complete pyloric obstruction secondary to peri-pyloric inflammation and edema. Histology in the siblings with co-existing Sarcina and H. pylori were nearly identical. C) Active ulcerating esophagitis (hematoxylin/eosin, ×200). D) Chronic active H. pylori gastritis (hematoxylin/eosin, ×400). E) Sarcina organisms in overlying gastric mucin showing characteristic basophilic tetrad packeted morphology (hematoxylin/eosin, ×600). F) H. pylori organisms in gastric pit (hematoxylin/eosin, ×400). H) pylori associated duodenitis was also present in both siblings (images not shown).

A previously healthy 12-year old male underwent esophagogastroduodenoscopy (EGD) following a 3-month history of emesis and epigastric pain, which did not respond to standard dose proton pump inhibitor. Endoscopy revealed erosive esophagitis in the distal third of the esophagus (Figure 1A), erythematous gastric mucosa and a highly edematous, tight pylorus (Figure 1B).At the time, serum antibodies for *H.pylori* were positive, and four months later, the patient’s 16-year old sister presented with similar symptoms. She had a history of medication-controlled gastroesophageal reflux disease with a normal EGD one-year prior; however, her current endoscopy was significant for severe erosive esophagitis and a highly edematous pylorus. In addition, the stomach was markedly inflamed and full of food debris. Endoscopic biopsies were obtained.

### Histopathology

Histologic features of the sibling’s biopsies were nearly identical: active erosive esophagitis with *Sarcina* present in ulcer bed debris; chronic active *H. pylori* gastritis with *Sarcina* and *H. pylori* organisms admixed in overlying mucin as well as in areas of erosion; and *H. pylori* duodenitis also with both organisms admixed in areas of erosion (Figures 1C–F). Classic tetrad packets of *Sarcina* organisms were present in both patients (Figure 1E). Although *H. Pylori* was present in surface mucin and gastric pits, *Sarcina* organisms were identified only in surface exudate and remained non-invasive in both patients.

### Polymerase chain reaction (PCR); DNA sequencing and alignment

Areas containing *Sarcina* organisms, present in formalin-fixed, paraffin-embedded tissue from biopsies of the brother’s esophagus and stomach and the sister’s esophageal biopsies were isolated through microdissection. The pyruvate decarboxylase (PDC) gene, present in only a few bacterial species, including *S. ventriculi*, allows bacteria to convert pyruvate to acetaldehyde and carbon dioxide, and was therefore used as positive identification. Exploiting this unique metabolic feature, primerswere designed (forward: 5′-AGCGGTTGCAGCGACAATAG-GA-3′; reverse: 5′-CTGCAACCAGCGCTGCACCT-3′) to target the PDC gene of *S.ventriculi* (AF354297.1), yielding a 149 base-pair amplicon. The PCR and DNA sequencing techniques used in this study follow the protocol outlined previously [8].

### Findings by DNA sequencing and alignment

Amplification of the PDC gene was present in DNA isolated from the brother’s esophagus and stomach biopsies, with a 96% blast match rate to *S. ventriculi*. Both of these samples, esophageal and stomach, matched to each other with 100% identity. Multiple attempts to amplify DNA isolated from the sister’s esophageal biopsy yielded no reaction. As positive control tissue produced expected results, the DNA from the sister’s sample was assumed to be non-viable.

## Discussion

The natural habitat of *Sarcina* is the soil, yet its presence has also been well documented in stagnant water, on the surface of cereal seeds, and interestingly as one of the major bacterial contaminants isolated from commercially available children’s soap bubbles [2–4,9].It has been postulated that when *Sarcina* is present in the human GI tract, the organisms are likely ingested with soil particles associated with food [3]. Further, *Sarcina* has been identified in the feces of healthy human adults, particularly vegetarians [1].

Although *Sarcina* is well-recognized in the veterinarian literature as a cause of abomasal gas and bloating in ruminants [10–13], its role as a causative agent of gastrointestinal disease versus bystander in humans remains uncertain. There have been a few reports of *Sarcina* causing acute emphysematous gastritis, a rare potentially fatal condition resulting from invasion of the gastric wall by gas-forming bacteria, including a three year-old child who recovered following antibiotic therapy against anaerobes [7].

*Sarcina* is not generally present in the normal healthy human stomach; however, it has been reported in the setting of pyloric ulceration and stenosis [3]. Indeed, when Dr. Ferrier of King’s College first documented the presence of *Sarcina* within the human stomach in 1842, he used the term “Sarcinous vomiting,” as he generally found the organisms in patients “with chronic diseases of the stomach associated with obstinate vomiting of acid, frothy, yeasty matters’ [2]. In the setting of gastric obstruction, the presence of carbohydrates and other nutrients in food provide fermentative substrates for *Sarcina* [3]. *Sarcina* thrives under these conditions since they are able to tolerate the strongly acidic environment of the stomach [3,11,14,15], while the growth of other microorganisms is inhibited at this low pH. In their recent review of endoscopic biopsies, Lam-Himlin and colleagues identified *Sarcina* most commonly in patients with a history of gastric outlet obstruction or delayed gastric emptying [8].

We describe pediatric siblings with identical presentations of active *H. pylori* gastritis/duodenitis with the co-existence of *Sarcina* occurring four months apart. Given their relationship, these two patients are assumed to have had similar exposures. The degree of gastric and duodenal inflammation and edema in both cases were marked enough to cause secondary gastric outlet obstruction including retained food and difficulty passing the endoscope through the pylorus. This presentation is in keeping with the documented association of the presence of *Sarcina* with gastric outlet obstruction.

In addition to the histologic evidence of *Sarcina* organisms, the brother’s esophagus and stomach biopsies showed the distinct 149 base-pair ampliconfrom the PDC gene of *S. ventriculi.* Subsequent sequencing showed a 96% homology (NCBI reference sequence AF354297.1) providing molecular confirmation of the morphologic impression of *S. ventriculi. Sarcina’s* characteristic histomorphology of basophilic tetrads or cubes of eight, measuring approximately the size of a red blood cell, make them quite distinct once recognized. Our experience with respect to these cases emphasizes that *Sarcina* organisms can be easily and accurately identified on H&E-stained sections. We speculate that these organisms are present in more cases of active esophagitis/gastritis than are currently documented and that increased awareness will lead to greater recognition.

## References

[1] Crowther JS (1971). Sarcina ventriculi in human faeces. J Med Microbiol.

[2] Ferrier D (1872). The Constant Occurrence of Sarcina Ventriculi (Goodsir) in the Blood of Man and the Lower Animals: With Remarks on the Nature of Sarcinous Vomiting. Br Med J.

[3] Canale-Parola E (1970). Biology of the sugar-fermenting Sarcinae. Bacteriol Rev.

[4] Willems A, Collins MD (1994). Phylogenetic placement of Sarcina ventriculi and Sarcina maxima within group I Clostridium, a possible problem for future revision of the genus Clostridium. Request for an opinion. Int J Sys Bacteriol.

[5] Holt SC, Canale-Parola E (1967). Fine Structure of Sarcina maxima and Sarcina ventriculi. J Bacteriol.

[6] Glass M (1973). Sarcina species on the skin of the human forearm. Trans St Johns Hosp Dermatol Soc.

[7] Laass MW, Pargac N, Fischer R, Bernhardt H, Knoke M (2010). Emphysematous gastritis caused by Sarcina ventriculi. Gastrointest Endosc.

[8] Lam-Himlin D, Tsiatis AC, Montgomery E, Pai RK, Brown AJ (2011). Sarcina organisms in the gastrointestinal tract: a clinicopathologic and molecular study. Am J Surg Pathol.

[9] McGarrity GJ, Coriell LL (1973). Bacterial contamination of children’s soap bubbles. Am J Dis Child.

[10] DeBey BM, Blanchard PC, Durfee PT (1996). Abomasal bloat associated with Sarcina-like bacteria in goat kids. J Am Vet Med Assoc.

[11] Vatn S, Tranulis MA, Hofshagen M (2000). Sarcina-like bacteria, Clostridium fallax and Clostridium sordellii in lambs with abomasal bloat, haemorrhage and ulcers. J Comp Pathol.

[12] Vatn S, Gunnes G, Nybø K, Juul HM (2000). Possible involvement of Sarcina ventriculi in canine and equine acute gastric dilatation. Acta Vet Scand.

[13] Edwards GT, Woodger NGA, Barlow AM, Bell SJ, Harwood DG (2008). Sarcina-like bacteria associated with bloat in young lambs and calves. Vet Rec.

[14] Lowe SE, Pankratz HS, Zeikus JG (1989). Influence of pH extremes on sporulation and ultrastructure of Sarcina ventriculi. J Bacteriol.

[15] Goodwin S, Zeikus JG (1987). Physiological adaptations of anaerobic bacteria to low pH: metabolic control of proton motive force in Sarcina ventriculi. J Bacteriol.

